# Myelopathy due to Spinal Extramedullary Hematopoiesis in a Patient with Polycythemia Vera

**DOI:** 10.1155/2017/2416365

**Published:** 2017-01-04

**Authors:** Shuhei Ito, Nobuyuki Fujita, Naobumi Hosogane, Narihito Nagoshi, Mitsuru Yagi, Akio Iwanami, Kota Watanabe, Takashi Tsuji, Masaya Nakamura, Morio Matsumoto, Ken Ishii

**Affiliations:** ^1^Department of Orthopaedic Surgery, Keio University School of Medicine, Tokyo, Japan; ^2^Department of Orthopaedic Surgery, National Defence Medical College, Saitama, Japan; ^3^Department of Orthopaedic Surgery, Fujita Health University School of Medicine, Aichi, Japan

## Abstract

Extramedullary hematopoiesis (EMH) occasionally occurs in patients exhibiting hematological disorders with decreased hematopoietic efficacy. EMH is rarely observed in the spinal epidural space and patients are usually asymptomatic. In particular, in the patients with polycythemia vera, spinal cord compression due to EMH is extremely rare. We report a case of polycythemia vera, in which operative therapy proved to be an effective treatment for myelopathy caused by spinal EMH.

## 1. Introduction

Extramedullary hematopoiesis (EMH) occasionally occurs in patients exhibiting hematological disorders with decreased hematopoietic efficacy, such as myelofibrosis, thalassemia, and polycythemia vera. The condition most commonly occurs at sites involved in embryonal hematopoiesis such as liver, spleen, and lymph nodes [1–3]. EMH is rarely observed in the spinal epidural space and patients are usually asymptomatic. Spinal cord compression due to EMH is extremely rare. The diagnosis relies on the history of hematological disorders, magnetic resonance imaging (MRI) findings of soft tissue masses that lead to spinal cord compression, and histological examination.

Polycythemia vera is a bone marrow disease marked by the excessive production of red blood cells and is occasionally accompanied by an increased number of white blood cells and platelets. We report a case of polycythemia vera with spinal cord compression caused by spinal EMH, in which operative therapy proved to be an effective treatment for myelopathy.

## 2. Case Presentation

**Figure 1:**
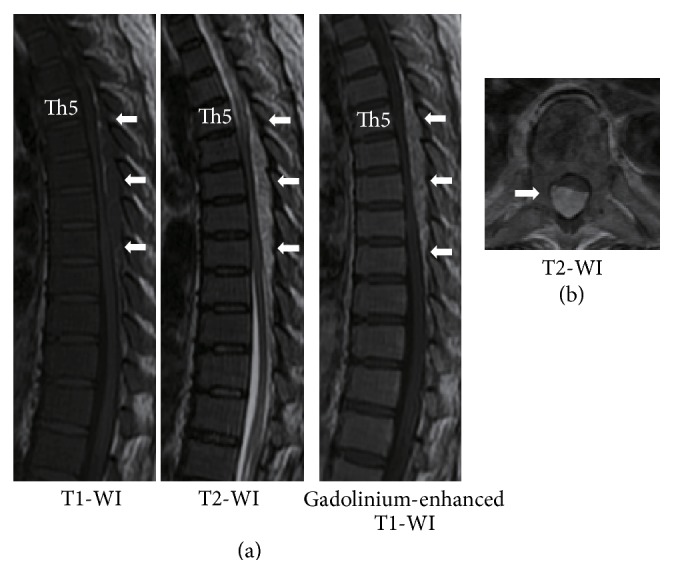
Preoperative MRI showed an epidural mass extending from the fifth to the tenth thoracic vertebra canal. (a) Sagittal plane. (b) Axial plane (Th7).

A 55-year-old woman had been diagnosed as having polycythemia vera in 2005 and followed up by a hematologist at another hospital. In 2012, she developed walking difficulty with progressive numbness and weakness in both legs, which started 3 months after radiation therapy for splenomegaly. She was seen at a nearby clinic, and MRI revealed an epidural lesion in the thoracic spine. At the initial visit to our hospital, cranial nerve examination and strength of the upper limbs were normal; however, both lower limbs were weak with power of 4/5 in iliopsoas, quadriceps, and hamstrings. Deep tendon reflexes were normal, and Babinski sign was negative. Sensation to touch and pain below navel was 5/10. The Japanese Orthopedic Association (JOA) score for thoracic myelopathy was 6/11. Blood test showed increased red blood cell count (19,900/*μ*L), hemoglobin level (17.6 g/dL), and reduced platelet count (74,000 *μ*L). Radiograph finding of the thoracic spine was normal. MRI of the thoracic spine showed an epidural mass extending from the fifth to the tenth thoracic vertebra (Figure 1). The lesion appeared isointense on T1-weighted images (WI) and hyperintense on T2-WI and showed heterogeneous enhancement after gadolinium administration. The spinal cord was compressed by the posterior epidural mass.

**Figure 2:**
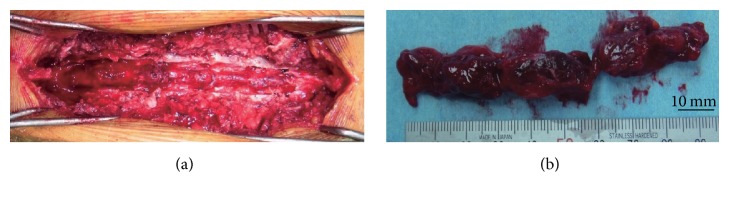
Photograph of the thoracic epidural mass. (a) The dorsal epidural mass is continuous from the fifth to the tenth thoracic vertebra canal after laminectomy. (b) The thoracic epidural mass specimen is reddish brown and hematoma like in appearance.

**Figure 3:**
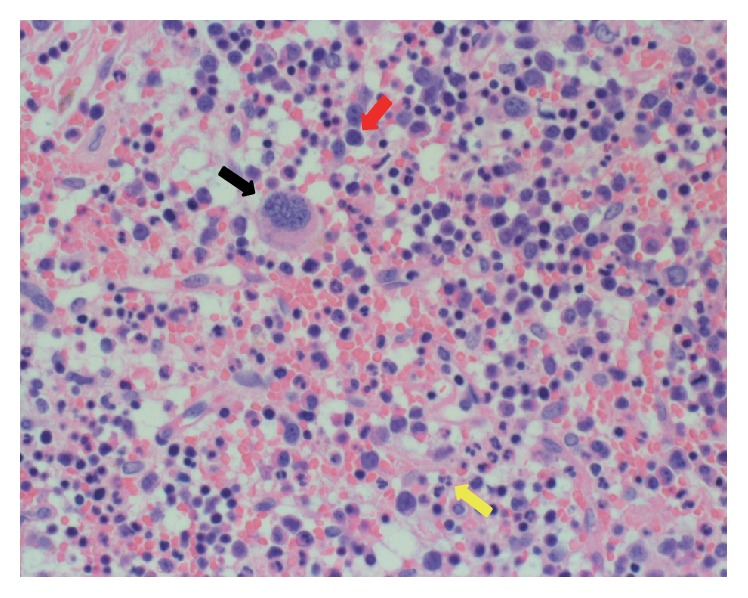
Histology of the epidural mass. Hematoxylin and eosin staining. Arrows suggest hematopoietic cells. Black, megakaryocytic; red, erythropoietic; yellow, myelopoietic. Magnification ×40.

**Figure 4:**
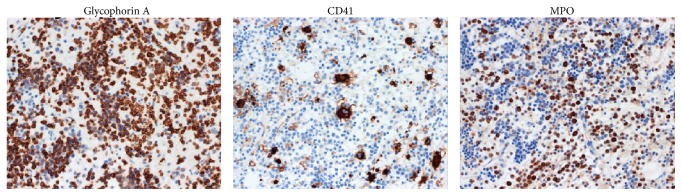
Immunohistochemistry of the epidural mass. High expression of cell surface markers for erythroblasts (glycophorin A), megakaryocytes (CD41), and granulocytes (MPO) was observed. Magnification ×40.

**Figure 5:**
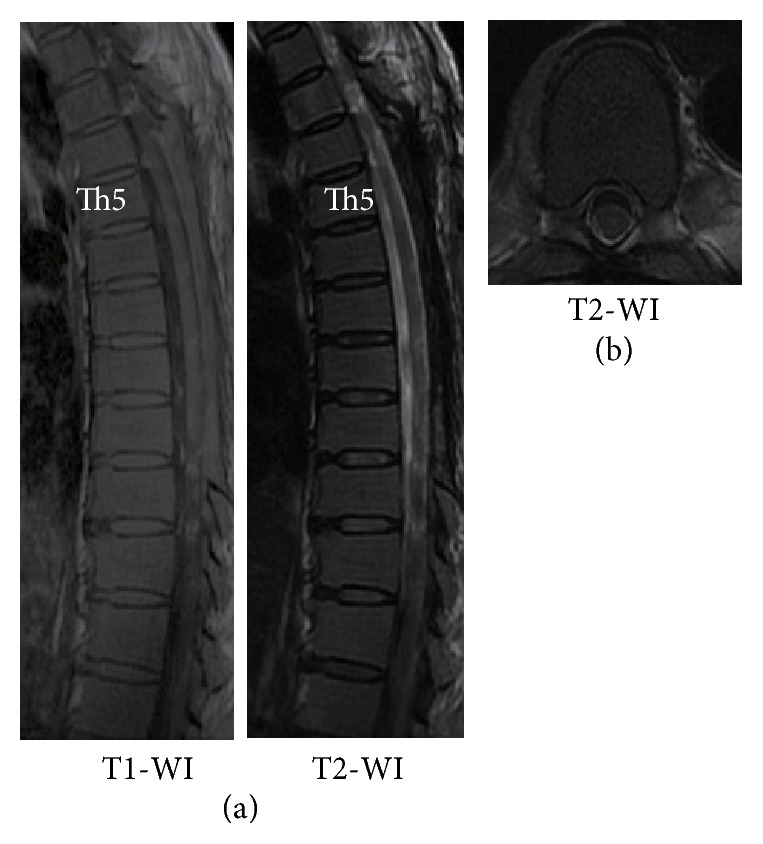
MRI taken one year later shows no recurrence. (a) Sagittal plane. (b) Axial plane.

Because her neurological symptoms were progressively getting worse, posterior decompression surgery was performed from the fourth to the ninth thoracic vertebra with intraoperative transfusion of platelets. The laminal arch of vertebrae from the fourth to the ninth thoracic spine was removed, and the reddish brown continuous mass was excised (Figure 2). The mass was not adhered to the dura mater and was easily removed. The operation time was 185 min, and the estimated blood loss was 1250 g. Histological examination confirmed the diagnosis of EMH because of the presence of hematopoietic cells differentiated into mature myelopoietic, erythropoietic, and megakaryocytic cells (Figure 3). Furthermore, immunohistochemical analysis of surgical sample was performed by using glycophorin A (DAKO, clone: JC159, 1 : 400) for erythroblasts, CD41 (Calbiochem, clone: 283.16B7, 1 : 2000) for megakaryocytes, and MPO (DAKO, rabbit polyclonal, 1 : 500) for granulocytes (Figure 4). These results also clearly supported the diagnosis of EMH. Immediately after surgery, she was able to walk and the leg numbness and weakness were resolved. She was not given postoperative radiation therapy and chemotherapy. A year after the surgery, there were no clinical or radiological signs of recurrence (Figure 5).

## 3. Discussion

Gatto et al. first reported a case of spinal EMH in 1954 [4]. Spinal lesions have been reported to occur in 11%–15% of EMH (male-to-female ratio of 2.5 : 1) and predominantly affect the thoracic spine [5–8]. Furthermore, 80% of all patients are asymptomatic; patients with myelopathy are uncommon [9]. In a study by Koch et al., spinal lesions of EMH were observed only in 0.6% of a total of 510 cases [10]. With respect to spinal cord compression due to spinal EMH, the most common underlying cause has been reported to be thalassemia according to a previous study of 42 patients [5, 6].

Polycythemia vera is a bone marrow disease marked by excessive production of red blood cells and is often accompanied by an increased number of white blood cells and platelets. The disorder is frequently observed in middle-aged and older men [11, 12]. The physical findings are nonspecific but may include an enlarged liver or spleen, plethora, or gouty nodules. It is often accompanied with circulatory disorders and coagulation abnormalities. Phlebotomy and chemotherapy are used to decrease blood thickness [13]. To the best of our knowledge, in polycythemia vera, myelopathy caused by spinal EMH is extremely rare with only 11 reported cases identified [12, 14, 15].

History of hematological disorders that could present with EMH is important for the diagnosis of spinal EMH. Moreover, MRI is useful as a diagnostic imaging modality. These masses appear as isointense signals on T1-WI and high-intensity signals on T2-WI and are often enhanced by gadolinium administration. Differential diagnosis includes lymphoma, metastatic spinal tumors, and epidural hematoma. A definitive diagnosis can be made by the identification of three hematopoietic cell elements on biopsy specimens or surgical samples.

The main treatment for myelopathy caused by spinal EMH is radiation or surgical decompression, and both therapies offer relatively good clinical outcomes [8, 16]. Although EMH has a relatively high radio-sensitivity and reduction of spinal lesions can be expected with the radiation, recurrence has been reported in some cases [17, 18]. In polycythemia vera, only two cases were previously reported to be treated by decompression surgery without radiation; however both of them showed no improvement of the symptoms [12]. On the other hand, in the present case, decompression surgery could achieve improvement of the symptoms without recurrence, indicating that surgical treatment may be also effective on the polycythemia vera patient with myelopathy due to spinal EMH. In cases of hematological disorders, including polycythemia vera, the platelet counts may be decreased; therefore, special care must be taken for perioperative bleeding when performing surgical treatment including laminectomy and excision of the EMH masses. In our case, intraoperative bleeding was 1250 g despite intraoperative transfusion of platelets. Previous reports have described surgical treatment combined with radiation therapy; however, in the present case, postoperative radiation therapy was not considered necessary as EMH masses were almost completely dissected during operation and the symptoms improved immediately after surgery. No recurrence was observed one year after the surgery as confirmed by MRI examination.

Taken together, occurrence of myelopathy and paralysis in patients with polycythemia vera should prompt investigators to confirm the presence of spinal EMH by immediate spinal MRI. If EMH is observed within the spinal column, a treatment strategy involving radiation therapy, surgery, or a combination of both should be considered. Patients with severe myelopathy or paralysis should be treated by early decompression surgery.
